# The Prognostic Role of Neutrophil Gelatinase-Associated Lipocalin and Kidney Injury Molecule-1 Expressions in Gastric Carcinomas

**DOI:** 10.3390/curroncol32040190

**Published:** 2025-03-25

**Authors:** Duygu Ayaz, Gülden Diniz, Ayşe Gül Pulular, Dudu Solakoğlu Kahraman, Umut Varol, Nuket Özkavruk Eliyatkın, Sevil Sayhan, Ali Kemal Kayapınar

**Affiliations:** 1 Department of Pathology, İzmir Faculty of Medicine, University of Health Sciences Turkey, İzmir Tepecik Education and Research Hospital, İzmir 35020, Turkey; dudu.solakoglukahraman@sbu.edu.tr (D.S.K.); sevil.sayhan@saglik.com.tr (S.S.); 2 Department of Pathology, İzmir Democracy University, Buca Seyfi Demirsoy Hospital, İzmir 35390, Turkey; gulden.diniz@idu.edu.tr (G.D.); aysegul.pulular@saglik.gov.tr (A.G.P.); 3 Department of Medical Oncology, İzmir Democracy University, Buca Seyfi Demirsoy Hospital, İzmir 35390, Turkey; umut.varol@idu.edu.tr; 4 Department of Pathology, Izmir Katip Çelebi University, Atatürk Education and Research Hospital, İzmir 35360, Turkey; nuket.ozkavruk.eliyatkin@ikcu.edu.tr; 5 Department of General Surgery, University of Health Sciences Turkey, Izmir City Hospital, İzmir 35540, Turkey; alikemal.kayapinar@saglik.gov.tr

**Keywords:** gastric carcinomas, NGAL, KIM-1, HAVcr-1, TIM-1

## Abstract

**Background:** The survival rate among stomach adenocarcinoma patients is exceedingly low. NGAL (neutrophil gelatinase-associated lipocalin) has pivotal roles in cell proliferation, immunity, and tumorigenesis. KIM-1 (Kidney Injury Molecule-1), also referred to as TIM-1 and HAVcr-1, is a transmembrane glycoprotein located in healthy immune cells and epithelial cells, and its upregulated form is generally found in several human cancers. **Aim:** The aim of this study was to investigate the prognostic significance of the expression of KIM-1 and NGAL in stomach cancers and identify NGAL-positive inflammatory cells in the tumor microenvironment. **Materials and Methods:** We immunohistochemically evaluated the expression of NGAL and KIM1 in 172 cases of stomach adenocarcinomas. **Result:** The mean age of the patients was 64.07 ± 12.35 years, and the mean and median follow-up period were 25.5 and 20.3 months, respectively. The expression rates of KIM-1 and NGAL in tumor cells were identical at 31.4% (n = 54). In 27 of these cases, both proteins were present. Among the deceased patients, the rate of simultaneous KIM-1 and NGAL positivity was relatively higher (*p* = 0.041). NGAL-positive inflammatory cells were observed in 13.4% of cases, with no significant correlation between these cells and survival times (*p* = 0.497). However, there was a negative correlation between survival times and KIM-1 (*p* = 0.037) and NGAL (*p* = 0.016) expressions in tumor cells. **Conclusions:** The present study has shown that KIM-1- and NGAL-positive tumor cells are influential in gastric tumorigenesis. Given the progress in anti-KIM-1 therapy, the presence of KIM-1 expression could contribute to the development of new treatment options for aggressive gastric cancer. However, these discoveries need to be validated in larger-scale studies.

## 1. Introduction

Among the most commonly encountered deadly cancers, stomach cancer ranks 5th by incidence and 4th by mortality rates worldwide [1,2]. Since overall five-year survival rates are very low, at the time of diagnosis, almost all cases have advanced stage unresectable tumors. Interactions among unique factors including environmental factors, diet, genetic predisposition, and *Helicobacter pylori (HP*) infection result in the advancement of gastric carcinoma [3,4,5].

Lipocalin 2 or 24p3, also known as neutrophil gelatinase-associated lipocalin (NGAL), is a part of the lipocalin superfamily. It was initially identified as a 25 kDa glycoprotein linked to matrix metalloproteinase-9 (MMP-9) in neutrophils [6]. NGAL was first classified as an acute phase protein due to its rapid release from neutrophils in response to tissue injury and inflammation [6,7]. Beyond its role in the acute phase response, NGAL is a critical component of the antimicrobial innate immune system, expressed by various hemopoietic cells [8]. For instance, granulocyte precursors and activated monocytes express NGAL, macrophages, neutrophils, and immature (CD34+) bone marrow progenitor cells. Contrarily, lymphocytes and plasmacytes do not express NGAL protein. The blood and urine of healthy people normally contain lower levels of NGAL produced by renal epithelial cells and neutrophils [6,7,8]. The expression of NGAL can play many physiological roles, regulating immune responses, such as transporting hydrophobic molecules across cell membranes, promoting epithelial-to-mesenchymal transitions (EMTs), and modulating iron metabolism. In summary, since NGAL performs various vital functions throughout various processes of development, tumorigenesis, and growth, it plays a role as a candidate marker for tumor growth in a fraction of solid tumors [8,9,10,11,12].

In 1996, a type 1 mucin-like membrane glycoprotein called kidney injury molecule-1 (KIM-1) was discovered. It is similar to proteins in the immunoglobulin family and facilitates the entrance of the hepatitis A virus into cells [11,12,13]. Therefore, it was once known as HAVcr-1, or the hepatitis A virus cellular receptor. After two years, this molecule was dubbed KIM-1 when it was found to be a very sensitive and specific marker for renal proximal tubule damage [11,12,13,14,15]. A family of proteins known as the TIM (T-cell immunoglobulin and mucin) domain family was identified in the 2000s; these proteins are mostly produced in respiratory system T cells. One of these proteins, TIM-1, is homologous to KIM-1. To sum up, in existing biology databases, the designations KIM-1, HAVcr-1, and TIM-1 (CD365) all refer to the same protein [16,17,18,19]. KIM-1 is normally expressed in the healthy kidney at a low level. However, cell-associated KIM-1 expression is enhanced considerably in the post-ischemic kidney, playing an anti-inflammatory role after kidney injury [11,12,13,14]. Furthermore, it has relatively little expression in healthy tissues and is increased in many human malignancies, including renal and ovarian carcinomas. As such, it is a prospective target for antibody-mediated treatment [15]. Additionally, kidney cancer can be detected early with the use of KIM-1, a blood-based marker [19]. CDX-O14 is a novel human monoclonal IgG1 antibody that is specially designed to target the extracellular region of TIM-1. The recombinant chimeric TIM-1-Fc protein and TIM-1 found on a variety of transformed cell lines are both efficiently bound by this antibody. However, it has not yet been incorporated into current treatment plans [17,18,19].

Thus far, many parameters like circulating tumor cells, microsatellite instability, HER2, PD-L1, and FOXP3 have been used as relevant markers to assess the tumor cell dynamics and proliferative activity of gastric malignancies. Still, a very limited number of tumor markers are being used in gastric carcinoma. For example, HER2- is a well-known prognostic biomarker whose expression is increased in gastric cancer. In addition, it has a role in both the neoadjuvant and metastatic settings. However, KIM-1 and NGAL have been seldom studied among these parameters [8,9,16]. This study sought to evaluate the significance of NGAL and KIM-1 expressions in stomach cancers.

## 2. Materials and Methods

We conducted a retrospective analysis of KIM-1 and NGAL protein expressions through immunohistochemical (IHC) staining on tissue specimens harvested from 172 primary stomach cancer patients that had undergone gastrectomy sometime between 2011 and 2018. The medical files of these cases were retrospectively evaluated. The local Ethics Committee of our research and training hospital approved this study (19.03.2015/2:20). HER2 expressions were also evaluated as a routine analysis in gastric cancer according to the guidelines released by the College of American Pathologists. We evaluated the hematoxylin-eosin (H&E)-stained archived slides again to identify the viable tumor areas and to select suitable paraffin blocks. The optimal areas for immunohistochemical evaluation were chosen and labeled first on the slide and then on the block. Then, paraffined cylindrical tissue samples of 2 mm in diameter were harvested from donor blocks. After the preparation of multiple tissue microarray (TMA) blocks using addressing and mapping techniques, we performed IHC using the diluted monoclonal rabbit antibodies against KIM-1 (Bioss, Philadelphia, PA, USA; HAVCRI) and NGAL (Novus Biologicals, Littleton, CO, USA; NDP1-90331) proteins at a dilution of 1:300. The pathologists, blinded to the patients’ clinical information, analyzed the slides and categorized the staining patterns based on their intensity.

**Figure 1:**
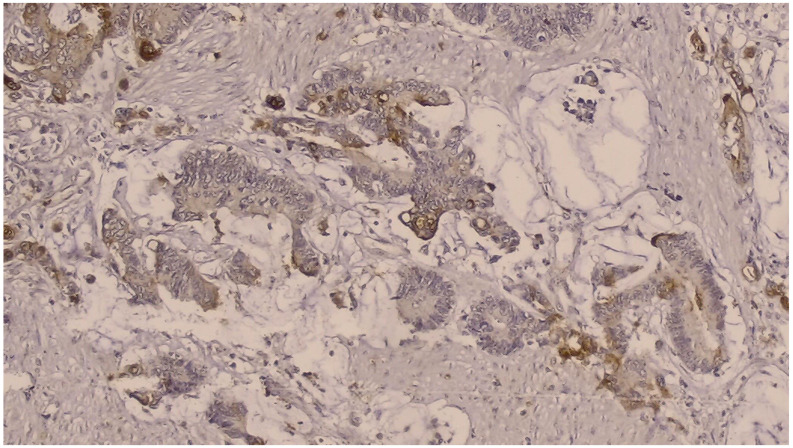
Immunohistochemically, cytoplasmic and a few intensely nuclear stained NGAL-positive tumor cells (DAB × 200).

**Figure 2:**
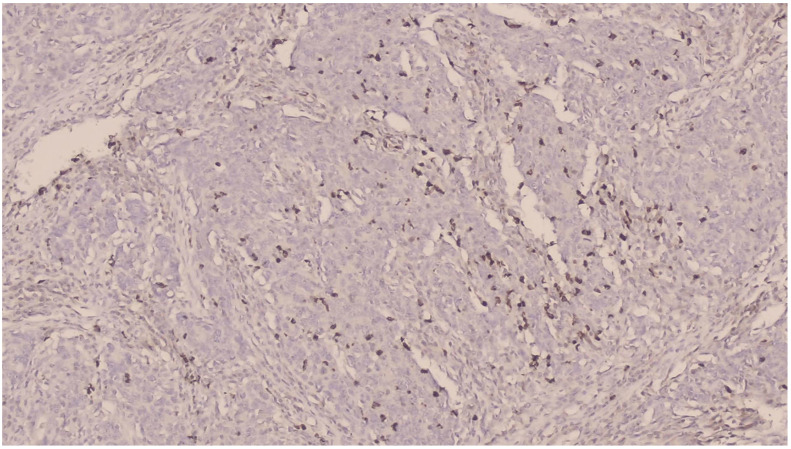
Note the NGAL-positive inflammatory cells in the tumor microenvironment (DAB × 200).

**Figure 3:**
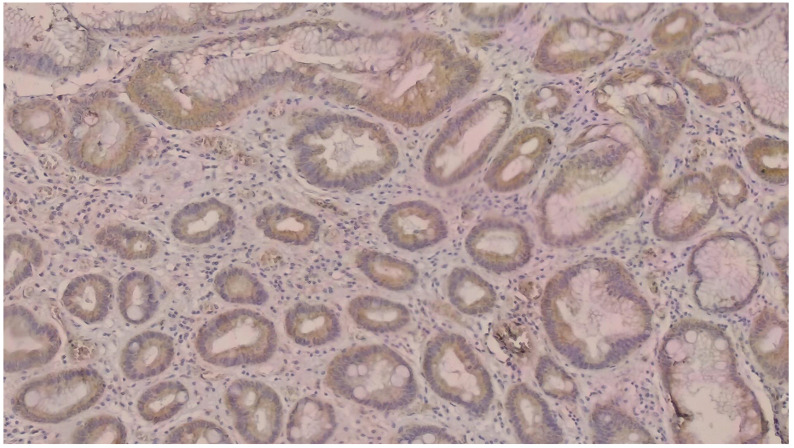
Immunohistochemically strong cytoplasmic KIM-1 positivity was determined in tumor cells (DAB × 200).

NGAL positivity was defined as diffuse or patchy areas of strong cytoplasmic and/or nuclear staining [Figure 1]. In addition, we counted the number of NGAL positive inflammatory cells that infiltrated the tumors per HPF, which was defined as NGAL positivity in the tumor microenvironment [Figure 2]. KIM-1 positivity was described as diffuse or patchy areas of strong cytoplasmic staining [Figure 3]. Since no tumors with nuclear NGAL expression alone were found, the tumors could not be grouped as nuclear and cytoplasmic expression. For both antibodies, strong focal staining or diffuse but very weak staining visible under a microscope was regarded as KIM-1 or NGAL negativity. Both antibodies were not scored because very weak staining was considered as negative.

The preferred software for statistical analysis was SPSS 25.0. The Mann–Whitney U test was applied to nonparametric data, while the chi-square test was utilized for the comparison of quantitative data. The nonparametric Kruskal–Wallis test was employed for comparisons among multiple groups, and Kaplan–Meier survival analysis was employed to rigorously assess survival rate differences between groups. A *p*-value of ≤0.05 was deemed statistically significant.

## 3. Results

In this series, 115 (66.9%) cases were male, and 57 (33.1%) were female. The average age of the 172 participants was 64.07 ± 12.3 years (median: 64 years; range: 29–92 years). The mean follow-up period was 25.5 ± 22.2 (0.07–85.8) months. The median follow-up period was 20.3 months. The demographic characteristics of the patients and results of the histopathological evaluation of the relevant slides are shown in Table 1. In this series, patient age (*p* = 0.023), presence of metastases (*p* = 0.003), tumor stage (*p* < 0.001), and the number of metastatic lymph nodes (*p* = 0.005) were found to have a statistically significant effect on survival rates.

According to the instructions, a combination of IHC and FISH methods were used. Finally, 29 cases (16.9%) were evaluated as HER2-positive, and all of them were prescribed appropriate anti-HER2 therapy (Table 2). Both HER2-negative and -positive tumors were found in the same location (*p* = 0.539). The death rate was higher in the HER2-positive group (65.5%) compared to the HER2-negative group (52.4%). However, there was no statistically significant difference between the survival rate and HER2 positivity (*p* = 0.197). The percentage of metastatic cases in the HER2-positive group (37.9%) was higher compared to the HER2-negative group (20.3%). The difference between HER2 positivity and the rate of metastasis was statistically significant (*p* = 0.037). The average age of patients and the diameter of HER2-positive and -negative tumors (64.25/63.17 years; 5.7/6.6 cm) were comparable without statistically significant differences between groups (*p* = 0.199, and *p* = 0.670, respectively). In addition, no statistically significant difference was seen between HER2-positive tumors in terms of many other clinicopathological parameters such as lymph node metastasis (*p* = 0.410), histological tumor type (*p* = 0.177), lymphatic vascular invasion (*p* = 0.179), and tumor stage (*p* = 0.821). Moreover, there was no statistically significant relationship between HER2 status and KIM-1 (*p* = 0.204) and NGAL (*p* = 0.628) positivity. It is noteworthy that the rate of perineural invasion was higher in HER2-negative tumors, and a significant negative correlation was seen between perineural invasion and HER2 positivity (*p* = 0.007).

**Figure 4:**
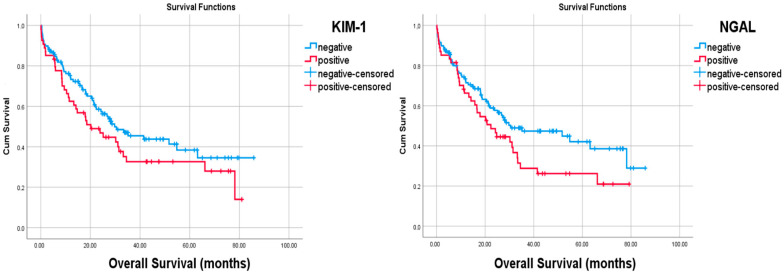
There were no statistical significances with overall survival and the presence of both KIM-1 (log Rank, *p* = 0.140) and NGAL (Log Rank, *p* = 0.78) expression by Kaplan–Meier survival analysis.

**Figure 5:**
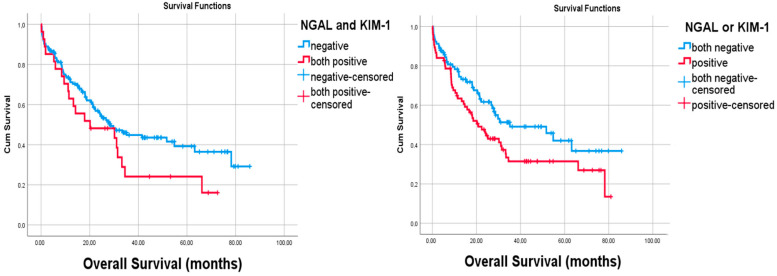
There were no statistical significances with overall survival and simultaneous expressions of both markers (log Rank, *p* = 0.127). Note the statistical significance between the absences of both markers and the overall survival (Log Rank, *p* = 0.043) by Kaplan–Meier survival analysis.

**Figure 6:**
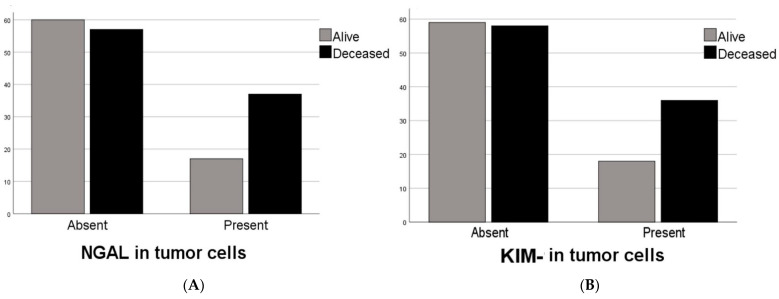
Note the negative relationship between the survival rate and the presence of (**A**) NGAL and (**B**) KIM-1 in tumor cells.

NGAL expression was observed in tumor cells of 54 (31.4%) cases. The number of KIM1-positive cases was also the same. Only 27 cases had both NGAL and KIM-1 immunoreactivity. Most of the clinicopathologic features such as overall survival (*p* = 0.978), tumor diameter (*p* = 0.319), tumor stage (*p* = 0.816), tumor location (*p* = 0.833), and the age (*p* = 0.888) and gender (*p* = 0.281) of these 27 cases did not differ when compared to NGAL- and KIM1-negative cases. The Kaplan–Meier survival analysis was performed with both the single marker and a combination of both markers, but statistical significance could not be shown [Figure 4]. On the other hand, there was a statistical significance with overall survivals and the absences of both markers (Log Rank, *p* = 0.043) by Kaplan–Meier Survival analysis [Figure 5]. Similarly, among the deceased patients, simultaneous KIM-1 and NGAL positivity rates were higher (*p* = 0.041). In addition, among the diffuse-type cancers (*p* = 0.027) and high-grade tumors (*p* = 0.021), the simultaneous KIM-1 and NGAL positivity rates were lower. NGAL-positive inflammatory cells were also observed within the tumors of 23 (13.4%) cases. However, KIM-1 positivity was not shown in inflammatory cells. Any significant correlation between the presence of NGAL-positive inflammatory cells and survival times (*p* = 0.497) was not detected, and there was no correlation between the presence of NGAL-positive inflammatory cells and tumor cells (*p* = 0.285). However, a negative correlation was found between the survival rates and the presence of KIM-1 expressions (*p* = 0.037) and the NGAL (*p* = 0.016) of tumor cells [Figure 6].

## 4. Discussion

A well-known signaling network exists between stromal and tumor cells, playing a crucial role in the formation of the tumor microenvironment. This microenvironment can affect cancer cell behavior in various ways, often promoting cancer progression. This environment consists of diverse cells from diverse origins that secrete various soluble factors such as microRNAs, growth factors, and cytokines. Adipocytes also secret NGAL whose main functions are the transportation of small hydrophobic molecules and the activation of innate immune responses. It was also discovered that NGAL secretion from the host adipose tissue surrounding the tumor can accelerate cancer progression by promoting EMT [20,21,22]. NGAL has dual roles in carcinogenesis, displaying both pro- and anti-tumoral effects depending on the type of cancer. Pro-tumoral effects include acting as an intracellular iron carrier and safeguarding MMP9 from proteolytic degradation, which has been observed in various cancers such as those of the breast, brain, uterine cervix, esophagus, and stomach. Additionally, NGAL is associated with NF-kB, a critical factor in tumor growth, neoplastic development, and chronic inflammation. Conversely, NGAL has been noted for its anti-metastatic and anti-tumoral properties in ovarian, pancreatic, and colon cancers. Some researchers suggest that NGAL can inhibit angiogenic factors like HIF-1 alpha and VEGF [23]. In addition, using a 3D spheroid model, it has been demonstrated that NGAL affects the early events in in vitro models of metastasis. The release of NGAL from macrophages increased local migration and invasion into the extracellular matrix and induced the epithelial–mesenchymal transition process in MCF-7 breast cancer cells. Accordingly, the role of NGAL released from macrophages in the progression of breast cancer was explored [6,7,8,9,10]. In this study, we also aimed to evaluate the potential use of NGAL levels for early diagnosis, prognosis prediction, and treatment response prediction and found a correlation between these parameters and survival rates in gastric carcinomas. More than half of the patients with NGAL-negative tumors survived, while most patients with NGAL-positive tumors died. However, any statistically significant correlation between NGAL-positive and -negative inflammatory cells in terms of clinicopathological features was not observed, which may be due to the presence of the small number of NGAL-positive inflammatory cells examined.

A recent study found that individuals with gastric cancer had different urine levels of NGAL compared to healthy control samples. Patients with gastric cancer had higher levels of the ADAM12 and MMP-9/NGAL complex in their urine [23]. Although early diagnosis of gastric cancer offers the chance for curative endoscopic resection, most patients may be diagnosed in advanced stages. Thus, there are no clinically useful noninvasive biomarkers for the early detection of gastric cancers. In the above-mentioned study, the authors claimed that urinary NGAL is a promising and easily detectable predictor for the early diagnosis of gastric cancer, because its levels increase in this type of cancer [23,24,25,26].

Similarly, its use as a diagnostic marker in luminal breast cancers has also been reported. By contrast, substantially decreased serum levels of NGAL have been detected in triple-negative and HER2-positive breast cancers [27,28]. However, we investigated NGAL positivity only in tumor tissue in our study. Therefore, we cannot comment on the urine and serum levels of NGAL in our patients, including their prognostic significance. In addition, since our study revealed that its expression in inflammatory cells did not have prognostic significance, we think that it may be more meaningful to immunohistochemically determine whether the increase in secretions of NGAL is caused by tumor cells.

Another study conducted by Kurozumi et al. revealed that the localization of NGAL is also associated with cancer outcomes. In their study, the low nuclear and high cytoplasmic localization of NGAL was related to the poor survival in breast cancer patients [29]. In our study, prominent nuclear staining without cytoplasmic NGAL expression was not determined. We determined mostly cytoplasmic expressions with or without nuclear staining in the tumor cells. Therefore, we cannot make conclusions regarding the relationship between survival rates and the location of NGAL expression.

Previous studies suggest that KIM-1’s phagocytic function helps clear apoptotic bodies in damaged proximal tubules, reducing antigen exposure to inflammatory cells and preventing excessive immune responses. However, when antigen-presenting cells (APCs) phagocytose apoptotic bodies, they activate regulatory cytotoxic T lymphocytes and T cells to attack target cells. Additionally, renal cell carcinomas (RCCs) arising from proximal tubules express KIM-1, indicating that some RCC cells may have phagocytic activity. It is proposed that RCC cells adopt KIM-1’s phagocytic function to engulf apoptotic bodies within tumors, thus avoiding the activation of T lymphocytes and APCs that would otherwise attack the RCC cells. Accordingly, KIM-1 acts as a scavenger for RCC cells, protecting them from immune reactions and aiding tumor development and survival.

One key indicator of clear-cell and papillary renal-cell carcinoma is the overexpression of KIM-1. The clinical significance of increased KIM-1 levels in extrarenal malignancies is still debated. Research by Lui et al. suggests that higher KIM-1 mRNA expression in gastric cancer is linked to a poor prognosis and reduced response to chemotherapy, leading to unfavorable outcomes. Zheng et al. found that elevated KIM-1 protein levels correlate with lower survival rates in non-small-cell lung cancer, indicating potential predictive value [17,18,19]. Additionally, in lung cancer, KIM-1 plays a crucial role in promoting invasion, proliferation, and migration, while the deactivation of KIM-1 leads to increased expression of the tumor suppressor PTEN and suppression of the pro-oncogenic PI3K/Akt pathway.

By contrast, Wang et al. discovered that KIM-1 mRNA overexpression in colon cancer tissues is associated with longer recurrence-free survival, indicating that the role of KIM-1 may vary depending on the tumor type and context. Additionally, elevated KIM-1 expression has been noted across a range of other cancers, including primary central nervous system lymphomas (54%), clear-cell ovarian carcinoma (93.8%), nephroblastomas (74%), endometrial carcinomas (33.3%), and germ cell tumors (50%). These findings suggest that KIM-1 expression can be both a marker of aggressive disease and a potential indicator of favorable outcomes, depending on the specific cancer type and biological context.

As a result, a clear-cut correlation between the KIM-1 expression level and the morphological and clinical characteristics of malignant diseases does not exist [11,13,14,15,16,17,18]. In this study, we have also investigated the potential use of KIM-1 expression as a prognostic factor for gastric carcinoma. Since the majority of patients with KIM-1-expressing tumors did not respond to conventional treatments and passed away, we decided to identify new immunomodulatory drugs that could potentially be used to treat patients with KIM gastric cancer. Considering KIM-1 as a new therapeutic target can help in designing and developing new drugs that can specifically inhibit the expression of KIM-1.

The present study showed that the expression of NGAL and KIM-1 may play a role in gastric carcinogenesis and can be useful as a biomarker in the diagnosis and tracking of gastric cancer, which in turn can allow for more accurate identification of patients at risk. Therefore, dependent on the progress of anti-KIM-1 treatments, targeting tumor cells expressing KIM-1 may be a new treatment option in gastric cancer. However, it is necessary to investigate and confirm these new findings in large-scale studies in patients with gastric carcinoma, so that our information about the role of these biomarkers in the diagnosis and more effective treatment of gastric cancer is more complete, so detailed and necessary instructions can be provided to doctors and patients about this new treatment.

## Figures and Tables

**Table 1:** Demographic, histopathologic, and immunohistochemical data of patients.

		n	%
**Prognosis**	Survived	78	45.3
	Exited	94	54.7
**Tumor Location**	Cardia/Proximal stomach	37	21.5
	Corpus/Distal stomach	135	78.5
**Diagnosis**	Intestinal-type	115	66.9
	Poorly adhesive/mixed	57	33.1
**Pathologic T stage**	pT1	12	7
	pT2	22	12.8
	pT3	79	45.9
	pT4	59	34.3
**Metastasis to local lymph node(s)**	Present	138	80.3
	Absent	34	19.7
**Distant Metastasis**	Liver	16	9.3
	Lungs	12	6.9
	Peritoneum	6	4.3
	Distant lymph nodes	4	2.3
	Ovaries	2	1.2
**Lymphovascular invasion**	Present	144	69.6
**Perineural invasion**	Present	104	75.3
**c-erbB2 expression** **(according to ASCO/CAP 2013 criteria)**	Negative or 1+	120	69.7
	2+	23	13.3
	3+/FISH positive	29	16.9
**NGAL expression**	Positive in tumor cell	54	31.4
	Positive in inflammatory cells	23	13.4
**KIM-1 expression**	Positive in tumor cell	54	31.4

**Table 2:** Association between clinicopathological findings of cases and HER2 positivity *.

HER2 Positivity (*)		Absent N/% N = 143/83.1%	Present N/% N = 29/16.89%	*p*
**Gender**	**Male**	95/33.6	20/69	0.792
	**Female**	48/33.6	9/31	
**Tumor location**	**Proximal Stomach**	32/22.4	5/17.2	0.529
	**Corpus/Distal**	111/77.6	24/82.8	
**Histological Type**	**Intestinal-type**	96/67.1	19/65.5	0.177
	**Poorly adhesive/mixed**	47/32.9	10/34.5	
**Survival status**	**Deceased**	75/52.4	19/65.5	0.197
**Lymph node metastases**	**Present**	116/81.2	22/75.9	0.410
**Location of distant metastases**	**Liver**	11/8.2	5/18.9	**0.037**
	**Lung**	11/8.2	1/3.8	
	**Peritoneum**	3/2.9	3/11.4	
	**Ovary**	1/0.9	1/3.8	
**Lymphovascular invasion**	**Present**	125/87.5	19/65.5	0.179
**Perineural invasion**	**Present**	90/62.9	14/48.2	**0.007**
**Tumor stage**	**Early**	28/19.6	7/24.1	0.821
	**Late**	115/80.4	22/75.9	
**KIM1 expression**	**Present**	42/29.4	12/41.4	0.204
**NGAL expression**	**Present**	46/32.2	9/27.6	0.628
**For Quantitative data**		**Mean ± SD**	**Mean ± SD**	*p*
	**Age (year)**	64.2 ± 12.7	63.2 ± 10.7	0.199
	**Tumor diameter (cm)**	5.7 ± 3.2	6.6 ± 3.6	0.670
	**Survival (months)**	25.6 ± 21.8	25.2 ± 24.3	0.197

## Data Availability

The data and materials used in this study are available upon reasonable request from the corresponding author (Ayaz D).
